# Shale gas reservoir characterization: A typical case in the Southeast Chongqing of Sichuan Basin, China

**DOI:** 10.1371/journal.pone.0199283

**Published:** 2018-06-27

**Authors:** Fangwen Chen, Shuangfang Lu, Xue Ding, Xipeng He

**Affiliations:** 1 Research Institute of Unconventional Oil & gas and New Energy, China University of Petroleum (East China), Qingdao, Shandong, China; 2 State Key Laboratory of Oil and Gas Reservoir Geology and Exploitation (Chengdu University of Technology), Chengdu, Sichuan, China; 3 Research Institute of Petroleum Exploration and Development, East China Branch, SINOPEC, Nanjing, Jiangsu, China; PLOS, UNITED KINGDOM

## Abstract

The Lower Silurian Longmaxi Shale in Southeast Chongqing of Sichuan Basin in China is considered to be a potential shale gas reservoir by many scholars in recent years. The special shale gas well, namely, Pengye-1 well, was selected as a case study to evaluate the characteristics of the shale gas reservoir. A series of experiments were performed to analyze the geochemical, mineralogical, and petrophysical features and gas content using samples of the Longmaxi Shale from Pengye-1 well. The results show that the organic and inorganic porosities of these samples are range of 0.08–2.73% and 0.06–2.65%, with the average of 1.10% and 1.76%, respectively. The inorganic pores primarily contribute to the porosity until the TOC content is more than 3%. Organic matter plays an important role in adsorbed gas content. The adsorbed gas is dominant in the Longmaxi Shale of Pengye-1 well, which ranges from 0.46 to 2.24 cm^3^/g, with an average of 1.38 cm^3^/g. The free gas content ranges from 0.45 to 0.84 cm^3^/g with an average of 0.68 cm^3^/g, and is 24.4–49.7 percent of total gas with an average of 37.5%. The bottom part of the Longmaxi Shale is the most favorable for shale gas exploring, which is higher of brittleness mineral content, porosity and gas content. Compare with the other five shales in America, the Lower Silurian Longmaxi Shale is derived from older sedimentary periods with significantly higher thermal maturity and has experienced several periods of intense tectonic, which are unfavorable for the shale gas enrichment.

## 1. Introduction

As a byproduct of economic and social development, energy demand has risen, and conventional oil and gas resources are constantly being consumed. The contradiction between the supply and demand of energy sources is become increasingly more serious, especially in China. Simultaneously, because of global climate warming, most countries and a variety of international environmental groups are calling for the use of clean energy resources and reducing emissions of greenhouse gases [1]. For these reasons, unconventional resources, particularly shale gas, have been receiving increasing attention.

America's "shale gas revolution" has greatly stimulated China to accelerate the pace of shale gas exploration and development. Preliminary evaluation shows that shale gas resources in China are as much as (15–30)×10^12^ m^3^, which is far more than that of conventional natural gas resources [2–5]. However, shale gas exploration in China cannot replicate the successful experience of the USA. The geological conditions of shale gas reservoirs in China are significantly different than those of the successful exploration areas in the USA. The marine shale gas reservoirs in southern China (i.e., the Lower Cambrian Niutitang shale and the Lower Silurian Longmaxi Shale) are derived from older sedimentary periods and have higher thermal maturity. They experienced strong multiphase tectonic movement, such as Indosinian, Yanshan and Himalayan period tectonic movements [6–9]. Shale gas development depends on the conditions of geochemical, geological and engineering technologies. Geochemical characteristics (organic matter abundance, type and maturity) and geological features (mineral composition, porosity and gas content) are the key parameters for shale gas reservoir evaluation [10–12] which determine the potential for shale gas production. Because of the high organic matter abundance, high thermal maturity and stratigraphic continuity, the Longmaxi Shale was selected in this paper as a case study shale gas reservoir.

The Lower Silurian Longmaxi Shale in the Southeast Chongqing has been regarded as a source rock in the past several decades and has become the focus target for shale gas exploration in recent years [13–16]. However, there are few wells that are drilled specifically for shale gas, and evaluations of shale gas reservoirs are limited. Pengye-1 well is a special cored well that was used to assess the geochemical and geological conditions of the Longmaxi Shale gas reservoir in 2012. Therefore, this paper investigates the Longmaxi Shale gas reservoir, especially the organic porosity, inorganic porosity and gas content, based on core samples from Pengye-1 well.

### 2. Geological setting

**Fig 1:**
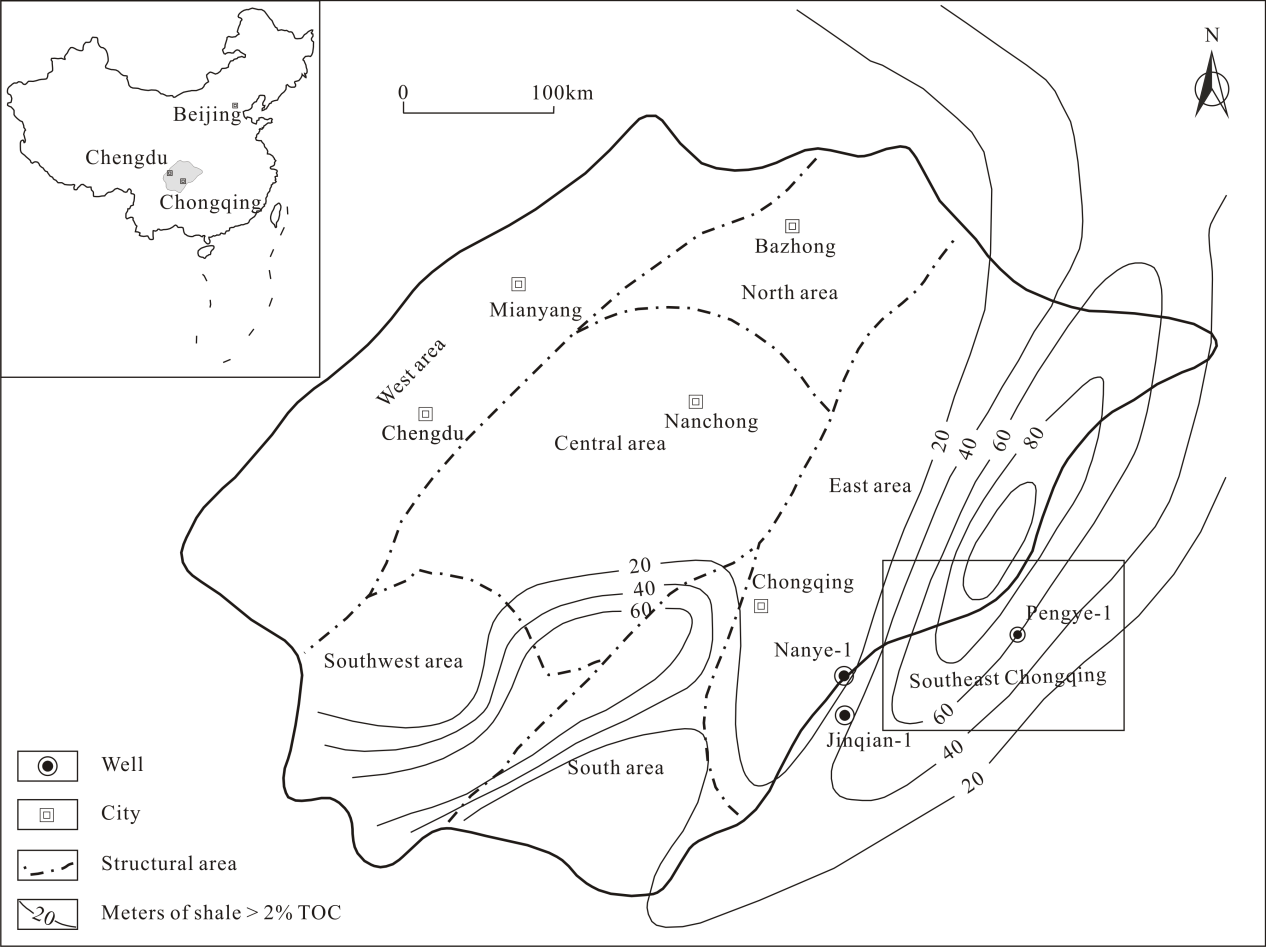
Stratigraphic column and isopach of the Longmaxi Shale in the Southeast Chongqing.

The Southeast Chongqing region investigated in this paper is located at the margin of the southeast Sichuan Basin. The Sichuan Basin, located in the west of the Yangtze metaplatform, is a large tectonically stable and old oil-gas-bearing superimposed basin, encompassing approximately 180 thousand square kilometers in Southwest China [17, 18]. The study area is in the southeast of Chongqing, China, and has an area of approximately 1.98×10^4^ km^2^ [19, 20] (Fig 1).

**Fig 2:**
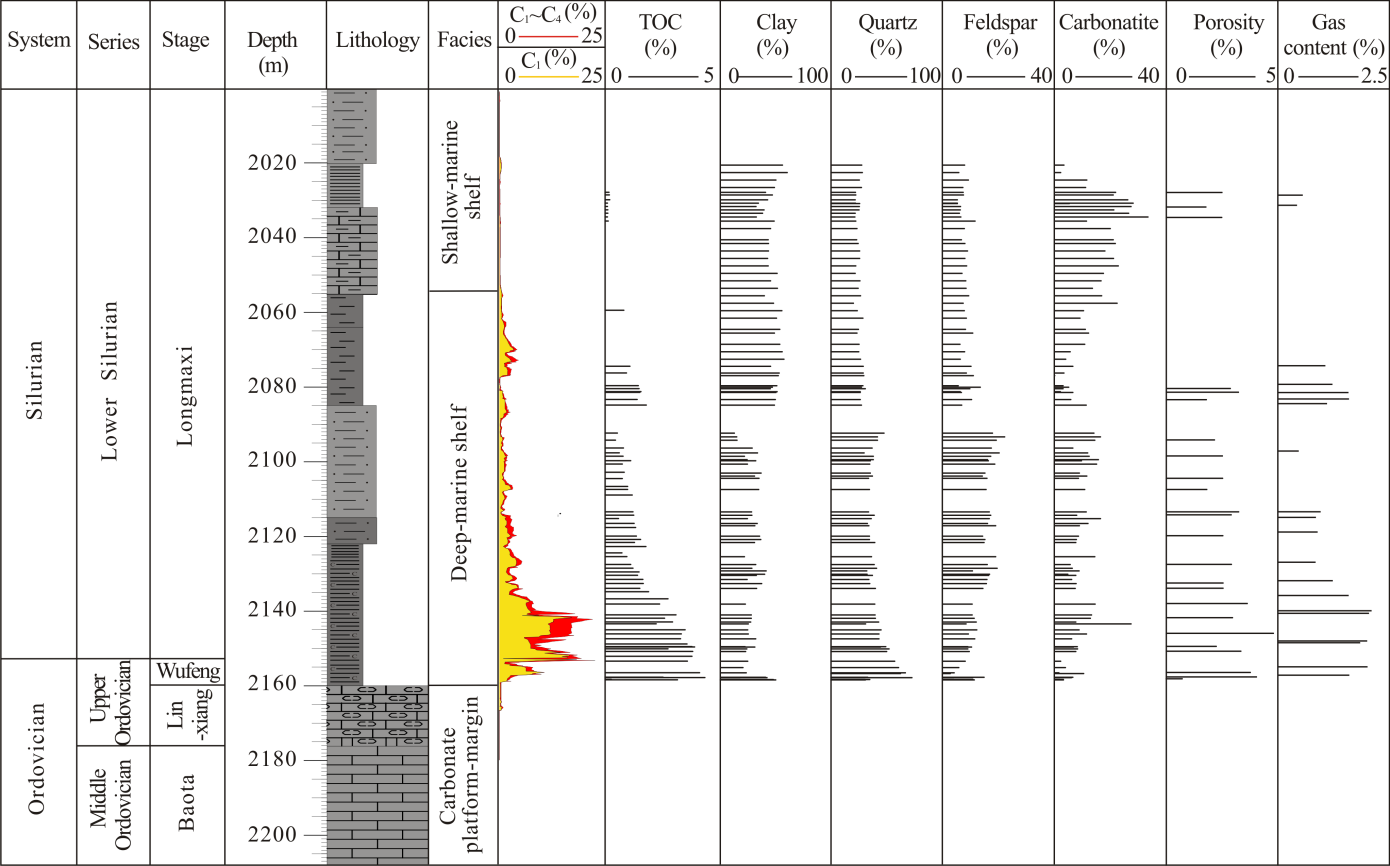
Characteristics of the Lower Silurian Longmaxi Shale in Pengye-1 well.

In the Southeast Chongqing region, the residual Paleozoic strata are of Cambrian, Ordovician and Silurian age, with other layers that are denuded or missing [19, 20]. The Lower Paleozoic marine shale, mainly including the Lower Cambrian Niutitang Shale and the Lower Silurian Longmaxi Shale, are widely deposited in the Southeast Chongqing. In the Longmaxi Shale, the bottom and middle parts are derived from a deep-marine shelf sedimentary environment, and the top part is derived from a shallow-marine shelf sedimentary environment [20, 21] (Fig 2).

There have been few special wells drilled to determine the characteristics of the Longmaxi Shale in shale gas research. Pengye-1 is an exploratory well that was specifically drilled to evaluate the Longmaxi Shale gas reservoir. At the bottom part of the Longmaxi Shale in Pengye-1 well (from 2160 m to 2122 m), grey and black carbonaceous shale and mudstone (approximately 38-m-thick) is present, including 8-m-thick carbonaceous shale of the Upper Ordovician Wufeng formation. In the middle part (from 2122 m to 2055 m), grey shaly sandstone (approximately 37-m-thick) and grey and black mudstone (approximately 30-m-thick) are found. In the top part (from 2055 m to 2010 m), grey lime mudstone (approximately 23-m-thick), shale (approximately 12-m-thick) and shaly sandstone are present (Fig 2). The formation pressure coefficient of the Longmaxi Shale reservoir in Pengye-1 well is 1, namely the reservoir pressure is equal to the hydrostatic pressure (about 21MPa). The temperature of target reservoir is 59–61°C via temperature logging.

## 3. Materials and methods

### 3.1 Samples

There are a few wells that had been drilled with the purpose of discovering shale gas and evaluating shale reservoir characteristics in the Lower Silurian Longmaxi formation of the Southeast Chongqing. This paper selected the core samples of Longmaxi Shale from Pengye-1 well (108°21’25”N, 29°18’59”E), which are representative of the great extent of the shale gas reservoir of interest. The location of the Pengye-1 well was permitted by Oil and Gas Resources Strategic Research Center, Ministry of Land and Resources of the People’s Republic of China. No endangered or protected species are involved in the field studies. The cores of Pengye-1 well were obtained using wire line coring in the drilling process with oil base mud. There are sixteen times of coring in the Longmaxi Formation with total drilling footage of 102.04 m. The total length of the cores is 92.09 m with an average recovery rate of 90.25%. The desorbed gas content measurements were conducted when the cores were obtained from the well hole. The core samples for measuring water saturation were sealed preservation. Most of the cores of Pengye-1 well are stored in the core library of East China Branch, SINOPEC.

### 3.2 Experiment

#### 3.2.1 TOC, kerogen type and maturity analysis

To estimate the shale gas reservoir, it is essential to confirm the various factors controlling the generation and storage of shale gas [10, 17]. Organic geochemistry is necessary to define the amount of gas generated from the shale layer. The key factors determining the volume of generated gas are the original total organic carbon (TOC), the kerogen type and the thermal maturity. In this paper, the geochemical parameters, including the evaluation of organic matter abundance (residual TOC), determination of organic matter type (kerogen optical maceral composition, H/C and O/C atomic ratios and δ^13^C), and description of thermal maturity (vitrinite reflectance), were measured. The residual TOC can be measured using a carbon and sulfur analyzer. The organic matter type can be assessed via transmitted light microscopy and the kerogen δ^13^C content, as measured by combustion module-CRDS for δ^13^C analysis. The vitrinite reflectance (*R*_o_) is the most common method to evaluate thermal maturity, which is implemented by a microscopic inspection of kerogen and analysis of the reflectivity of the particles via a photomultiplier.

#### 3.2.2 XRD analysis

The mineralogical compositions of the samples were determined based on XRD patterns measured on randomly oriented powder preparates using an Ultima IV X-ray diffractometer at 40 kV and 30 mA with Cu Kα radiation (λ = 1.5406 for Cu Kα1). The crystalline mineral proportions (wt %) were calculated based on the areas under the peaks corresponding to each mineral and corrected using Lorentz Polarization [22]. It not only offers a quantitative evaluation of the crystalline components but also relates to the petrophysical properties and the friability of the shale.

#### 3.2.3 Total porosity and specific surface area

To assess the shale gas reservoir, it is also important to discuss its total porosity. This paper adopted the current energy industry standard porosity measurement protocol from gas shale samples based on a helium pycnometry technique proposed by the Gas Research Institute (GRI) [23, 24]. Shale samples with a mass of approximately 250 g were prepared for the total porosity method. The samples were preheated in a vacuum oven at 100°C for 12–16 hours to remove all pore fluids, such as water and oil. Afterwards, each sample was weighed using a balance set-up (Mettler Toledo AL104, readability 0.1 mg), and the bulk volume was measured by mercury immersion at less than 6.6 Pa (50 μm Hg) according to Archimedes’ Principle. The bulk density (*ρ*_b_) was calculated as the bulk volume divided by the bulk weight. Then, the sample was crushed into 80–200 mesh; after obtaining the grain weight, the grain volume was measured by helium pycnometry and Boyle’s law. The grain density (*ρ*_g_) was calculated using the grain volume divided by the grain weight. The total porosity (*Ф*_total_) was calculated using the Formula (1) (dx.doi.org/10.17504/protocols.io.icycaxw).

$$\Phi_{total}=\frac{\rho_{g}-\rho_{b}}{\rho_{g}}\times 100\%$$ (1)

#### 3.2.4 Low temperature nitrogen adsorption

The low temperature nitrogen adsorption was performed using a Micromeritics ASAP 2020 Surface Area and Porosity Analyzer. Powder samples of approximately 500 mg each (60–80 mesh grain size was chosen for consistency) were preheated in a vacuum oven of 10 μm Hg at 80°C for 12–16 hours to remove all fluids (water, oil) and adsorbed gas in pores. Reagent grade nitrogen (99.999%) was used as an adsorbate at -196.15°C, and adsorption/desorption isotherms were determined by recording adsorbed nitrogen quantities during the increase and decrease of relative pressures (*P*/*P*^0^) ranging between 0.01–1 with the equilibration time set to 10 s. The specific surface areas of these samples were calculated using the Brunauer Emmette Teller (BET) and Langmuir models.

#### 3.2.5 Measurements of gas content

The amount of shale gas is a key parameter for shale gas reservoir analysis. This paper used three methods to evaluate the amount and storage capacity of shale gas: gas logging, desorbed gas content measurement and isothermal adsorption.

Gas logging can be used to qualitatively evaluate the gas bearing characteristics of a shale gas reservoir. This technology, using a degasser, obtains gas carried by drilling fluid and detects the composition and content of gases, especially total hydrocarbon gas logging and methane logging. Generally speaking, higher methane logging values indicate a greater amount shale gas.

The isothermal adsorption experiment can evaluate the sorption gas capacity of shale samples as a function of pressure and temperature based on monolayer absorption. The experiments were performed at the shale gas reservoir temperature and pressure. The parameters of the Langmuir isotherm, such as the Langmuir volume (*V*_L_) and the Langmuir pressure (*P*_L_), were calculated by fitting the experimental results [25].

The desorbed gas content measurement consists of three parts [26, 27]: (1) desorbed gas measurement, which directly measures the volume of shale gas released from a core sample sealed into a desorption canister; (2) residual gas measurement, which directly measures the volume of shale gas remaining in a core sample after the expiration of desorbed gas measurement; and (3) lost gas estimation, which indirectly estimates the volume of the gas desorbed from the core sample during its collection and before being sealed into an airtight desorption canister based on empirical correlations and gas storage capacity data derived from an isothermal adsorption experiment. This method was developed by the former USBM (US Bureau of Mines) [28] from the research of Bertard [29].

## 4. Results and discussion

### 4.1 Geochemical characterization

A series of geochemical tests were implemented in core samples representing approximately 160 m of the Lower Silurian Longmaxi Shale, especially in the bottom and middle parts. A relatively complete geochemical characteristic profile of the Longmaxi Shale in Pengye-1 well was developed.

### 4.1.1 Organic matter abundance

The TOC values from a total of 72 samples range from 0.11% to 4.34%, with an average of 1.68%. The TOC decreases from the bottom to the top in the Longmaxi Shale of Pengye-1 well (Fig 2). In the bottom part, the TOC values from 33 samples range from 0.73% to 4.34%, with an average of 2.66%. In the middle part, the TOC values from 29 samples range from 0.46% to 1.80%, with an average of 1.09%. In the top part, the TOC values from 10 samples range from 0.11% to 0.27%, with an average value of 0.16%. The TOC values of 22 samples are greater than 2.0%, with an average of 3.29%. These samples are all distributed in a 23-m-thick carbonaceous shale section at the bottom of the Longmaxi Shale.

### 4.1.2 Organic matter type

The maceral composition, H/C and O/C ratios, infrared spectrum, rock pyrolysis and kerogen δ^13^C content are commonly used to classify the source rock type [30, 31]. According to these parameters, the organic matter can be divided into four types: sapropelic (I), humic-sapropelic (II_1_), sapropelic-humic (II_2_) and humic (III) [32, 33] (Table 1). However, for high thermal maturity source rock H/C and O/C ratio analyses, the infrared spectrum and rock pyrolysis are not feasible or accurate for classifying the source rock type [30, 34]. Because of the high thermal maturity of the Longmaxi Shale, this paper selected the kerogen δ^13^C content and maceral composition to clarify the type of Longmaxi Shale in the study area.

**Table 1:** Organic matter type classified by the kerogen δ^13^C content and maceral composition.

Organic matter type	Kerogen δ^13^C content (‰)	Maceral of kerogen (TI value)
Sapropelic (I)	<-29	>80
Humic-sapropilic (II_1_)	-29~-27	40–80
Sapropilic-humic (II_2_)	-27~-25	0–40
Humic (III)	>-25	<0

The kerogen δ^13^C content values from 2 samples of Pengye-1 well are -29.78‰ and -30.04‰. The source rock organic maceral were mainly animal organic matter, secondary components and mineral-bituminous matrix, and vitrinite, inertinite, exinite and sapropelite were not detected. Secondary components consisted of micrinite and solid bitumen (Table 2). Some studies of the organic matter of the Longmaxi Shale have been performed in the Southeast Chongqing and its adjacent areas. The kerogen δ^13^C content of the Longmaxi Shale in the Southeast Chongqing and its adjacent areas has been reported by many scholars: -30.51‰ and -29.67‰ from the Jianshen-1 well in eastern Chongqing [35]; three basset sample values between -29.26 ‰ and -29.55‰, with an average of -29.43‰ in the Southeast Chongqing [36]; ten basset sample values between -27.72 ‰ and -30.83‰, with an average of -29.11‰ in eastern Chongqing [37]; and between -28.78‰ and -32.04‰, with an average of -30.23‰ in the Sichuan Basin [38]. Based on these data, the organic type is I. Furthermore, other studies have also demonstrated the organic matter type is I using the maceral composition [31, 37]. Therefore, comprehensive analyses in pre-existing studies suggest that the organic matter type of the Longmaxi Shale is sapropelic (I), which has a strong capability of generating oil.

**Table 2:** Maceral composition (vol. %), mineral matter content (vol. %) and vitrinite reflectance equivalence (*R*_o_ %).

Depth	Animal organic matter		Secondary components		Mineral-bituminous matrix	Mineral matter	Vitrinite reflectance equivalence
	Graptolite	Chitinozoa	Solid bitumen	Micrinite			
2080.42	0.75	0.25	0.13	0.50	50.13	48.24	2.70
2083.36	0.25	/	0.13	1.13	39.13	59.36	2.78
2094.20	0.25	/	0.13	0.63	8.75	90.24	2.54
2099.70	0.63	0.25	0.38	0.75	14.13	83.86	2.48
2107.45	0.13	0.13	0.25	1.25	11.75	86.49	2.31
2113.46	0.38	0.13	0.25	1.25	17.88	80.11	2.81
2132.61	0.63	0.13	0.25	2.13	27.13	69.73	2.66
2138.13	0.38	0.13	0.38	1.63	28.75	68.73	2.76
2141.89	0.38	/	0.50	1.75	34.50	62.87	2.50
2146.13	0.50	0.13	/	2.13	41.88	55.36	2.81
2149.49	1.13	/	/	2.38	42.75	53.74	2.64
2156.55	0.75	/	2.63	9.51	43.43	43.68	2.57
2157.76	0.38	/	0.38	3.88	45.63	49.73	2.79

### 4.1.3 Thermal maturity

The thermal maturity of the source rock was measured in 13 samples using microscopic inspection. The parameter of bitumen reflectance (*R*_b_), also called marine vitrinite, is derived from the marine sediment in the Early Paleozoic strata. The relationship between vitrinite reflectance *R*_o_ and *R*_b_ has been previously established [39, 40]. *R*_o_ was calculated using the following formula:

$$R_{o}=0.618R_{b}+0.4$$ (2)

Using the above formula, the bitumen reflectance (*R*_b_) was transformed into equivalent vitrinite reflectance (*R*_o_), which was used for shale gas evaluation. The equivalent vitrinite reflectance values were in the range of 2.31% to 2.81, with an average value of 2.64%, which indicated that the Longmaxi Shale in study area was in the over-mature stage. This reflects that the Longmaxi Shale is in the dry gas generation window. In the over-mature thermal stage, source rock mainly generates dry gas accompanied with a small amount of gas condensate [17].

Research has shown that large quantities of gas have been generated, especially from the secondary cracking of in-situ oils in the thermally over-mature stage [41, 42]. With reference to the Barnett shale, the primarily source of gas in the prolific Newark East Field is considered to be from the secondary cracking of oil and bitumen [12, 43]. Therefore, the Longmaxi Shale has favorable shale gas potential in the Southeast Chongqing due to its large organic matter abundance, sapropelic type and high thermal maturity.

### 4.2 Mineralogical characterization

This paper used the XRD technique to quantitatively analyze 83 samples in the Longmaxi Shale from Pengye-1 well. The interval of these samples is a short distance of approximately 2 m to fully represent the characteristics of the Longmaxi Shale mineral composition. The analysis results show that clay, quartz, feldspar and carbonatite (calcite and dolomite) are the main mineral compositions of the Longmaxi Shale, with average contents of 37.86% (range of 13.0%-60.8%), 33.66% (range of 20.4%-73.1%), 10.85% (range of 4%-20.3%), 11.72% (range of 1.7%-33.6%), respectively. The other mineral compositions such as gypsum, pyrite, siderite and barite are less than 5% in total content. It is indicated that all samples contain clay minerals composed of illite, chlorite and an illite-smectite mixed layer. Illite has the highest clay mineral content, ranging from 7.7% to 23.18%, with a mean of 15.01%. The other clay minerals, such as chlorite and the illite-smectite mixed layer, are in the ranges of 1.16% to 9.78% and 5.22% to 25.19%, with average contents of 3.98% and 14.3%, respectively. In the Longmaxi Shale from Pengye-1 well, the clay mineral content (non-brittle mineral) increases with increasing burial depth, whereas the quartz content (brittle minerals) decrease with increasing burial depth, especially at the bottom of the Longmaxi Shale. The average clay and quartz content at the bottom of the Longmaxi Shale are 29.58% and 43.64%, respectively.

Clay minerals play a critical role in shale gas reservoirs. Clay minerals can form a “cardhouse” structure of individual edge-face- or edge-edge-oriented flakes and/or domains of face-face-oriented flakes [44–46]. The volume of this structure is composed of interparticle clay flake pores. Although these pores are primarily found in deeply buried ancient shale, how this cardhouse pattern has survived burial and diagenesis for hundreds of millions of years remains unknown [47]. Pores between floccules are larger than the diameter of methane molecules (0.38 nm) and may be interconnected to form permeability pathways [46]. However, higher clay mineral contents lead to a lower brittleness of shale gas reservoirs [48]. Shale formations with higher clay mineral content are not favorable for horizontal well fracturing operation, whereas hydraulic fracturing is necessary for shale gas wells. Considering the mineral composition, the bottom part is the most favorable for hydraulic fracturing to produce shale gas in the Longmaxi Shale.

### 4.3 Porosity and specific surface area

The porosity of shale samples impacts the capacity of shale gas, especially the free gas potential [48]. The free gas potential and adsorbed gas capacity indicate the maximum potential shale gas capacity, which is used to evaluate the commercial value of the shale gas reservoir [12]. The results of GRI method showed that the total porosity ranges from 1.79% to 4.82%, with an average of 2.85% for 23 cored samples from Pengye-1 well (Fig 2 and Table 3). The porosities were divided into organic and inorganic porosity. The organic pores are main nano-scale pores within organic matter grain formed from hydrocarbon generation. The organic porosity was calculated using the material balance principle and corrected by the organic pore correction coefficient (Formula 3), which was introduced in previous research results [49] (dx.doi.org/10.17504/protocols.io.iczcax6). The inorganic pores mainly occur within inorganic matrix of shale, including porous floccules, interparticle pores, microchannels, fractures and intraparticle pores located within mineral particles [46]. The inorganic porosity is the different of total porosity and organic porosity. The organic and inorganic porosities of these samples are range of 0.08–2.73% and 0.06–2.65%, with the average of 1.10% and 1.76%, respectively (Table 3). The total porosity contains organic porosity and inorganic porosity, and it also reflects the connected and unconnected pore volumes. The inorganic pores have a higher probability of being part of an effective pore network than the organic pores in the Longmaxi Shale from Pengye-1 well, because the inorganic pores are main interparticle pores and the organic pores are main intraparticle pores.

$$\Phi_{\text{organic}}=w({\text{TOC}}_{0})\cdot I_{\text{H0}}\cdot F(R_{o})\cdot \frac{\rho_{rock}}{\rho_{kerogen}}/1000\cdot C$$ (3)

where *Ф*_organic_ is the organic porosity of shale (%), *w*(TOC_0_) is the weight percent of the original total organic carbon (%), *I*_H0_ is the original cracking hydrocarbon of unit quality organic carbon (mg/g), *F*(*R*_o_) is the transformation ratio of oil and gas generated from organic matter (%), which is correlated with maturity, *ρ*_rock_ is the density of shale (g/cm^3^), *ρ*_kerogen_ is the density of kerogen, which is approximately 1.2 g/cm^3^, and *C* is the organic pore correction coefficient.

**Table 3:** Porosities and specific surface areas of the Longmaxi Shale samples from Pengye-1 well.

Depth (m)	TOC (%)	Grain density (g/cm^3^)	Bulk density (g/cm^3^)	Total porosity (%)	Organic Porosity ^a^ (%)	Inorganic Porosity ^b^ (%)	Sepcific surface area (m^2^/g)	
							Langmuir	BET
2027.78	0.17	2.78	2.71	2.52	0.11	2.41	7.2	9.9
2031.65	0.11	2.79	2.74	1.79	0.08	1.71	5.6	7.7
2034.45	0.11	2.79	2.72	2.51	0.08	2.43	5.4	7.3
2080.42	1.48	2.77	2.69	2.89	0.97	1.92	15.9	21.5
2081.44	1.51	2.77	2.68	3.25	0.99	2.26	17.0	23.0
2083.35	1.4	2.76	2.71	1.81	0.93	0.88	16.6	22.5
2094.2	0.46	2.75	2.69	2.18	0.32	1.86	/	/
2098.47	0.78	2.76	2.69	2.54	0.53	2.01	/	/
2104.46	0.76	2.75	2.68	2.55	0.58	1.97	8.4	11.4
2107.45	0.99	2.73	2.68	1.83	0.65	1.18	10.2	13.9
2113.46	1.21	2.76	2.67	3.26	0.81	2.45	10.1	13.8
2114.29	1.25	2.73	2.65	2.93	0.82	2.11	10.9	14.8
2119.86	1.37	2.74	2.67	2.55	1.02	1.53	11.9	16.2
2127.55	1.12	2.72	2.64	2.94	0.78	2.16	10.2	13.8
2132.6	1.66	2.73	2.66	2.56	1.09	1.47	12.9	17.5
2133.95	1.51	2.73	2.66	2.56	0.98	1.58	12.5	17.0
2138.13	2.38	2.74	2.64	3.65	1.54	2.11	15.9	21.6
2141.92	2.59	2.68	2.6	2.99	1.70	1.29	17.5	23.7
2146.13	3.32	2.7	2.57	4.81	2.16	2.65	18.1	24.5
2149.6	3.9	2.66	2.6	2.26	2.20	0.06	20.5	27.9
2150.84	3.81	2.68	2.59	3.36	2.49	0.87	19.6	26.4
2156.55	4.12	2.64	2.54	3.79	2.73	1.06	20.0	27.3
2157.75	2.52	2.71	2.6	4.06	1.63	2.43	23.3	31.5

^a^ The organic porosities were from Chen et al., 2014 [49].

^b^ The inorganic porosities were the difference between total porosity and organic porosity.

**Fig 3:**
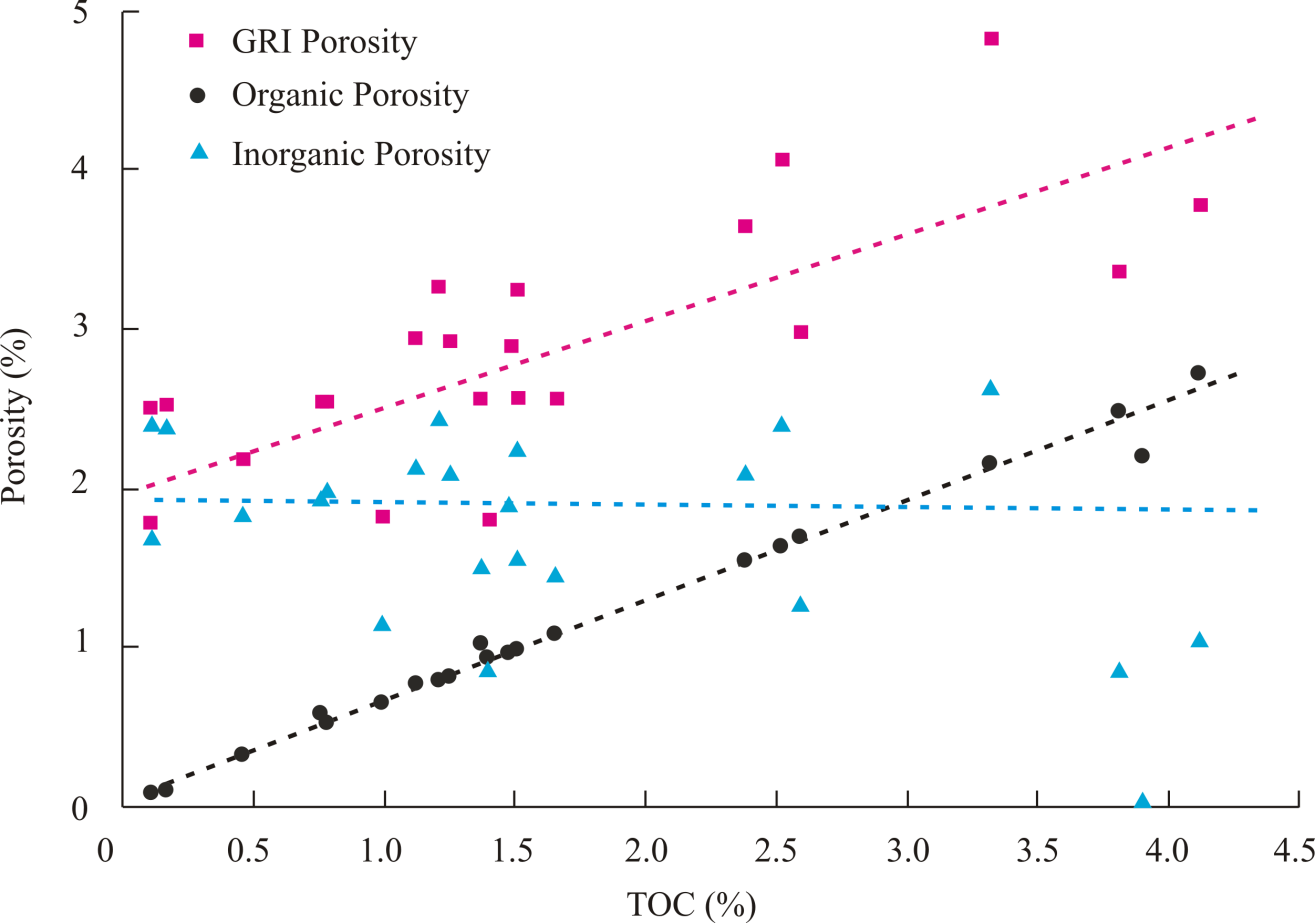
The relationship between TOC content and porosities of samples from Pengye-1 well.

Generally, the porosity of the reservoir decreases with increasing burial depth due to the process of compaction and diagenesis. However, the porosities of Longmaxi Shale have an obvious positive correlation with the TOC content (Fig 3), and there were no significant relationships with other minerals, such as clay, quartz, feldspar and carbonate. This is due to the inorganic porosities of shale samples with different TOC content are almost equal, while the organic porosities of these samples obviously increase with increasing of TOC content. The inorganic pores primarily contribute to the porosity until the TOC content is more than 3%.

**Fig 4:**
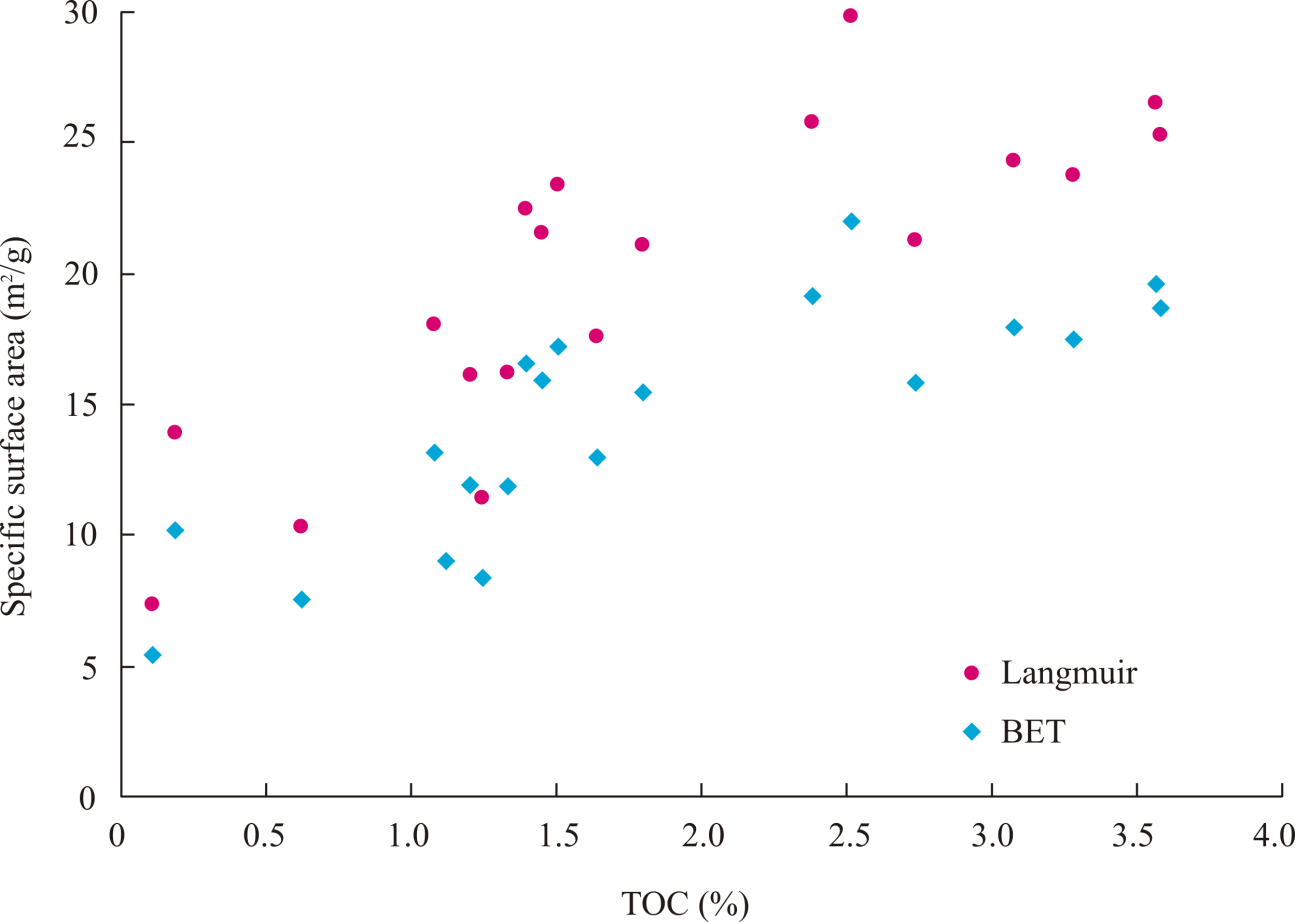
The relationship between TOC and specific surface area of samples from Pengye-1 well.

The specific surface areas of these samples calculated using the Langmuir and Brunauer Emmette Teller (BET) models from were listed in Table 3. The Langmuir and BET specific surface areas of these samples are in the range of 7.3–31.5 m^2^/g and 5.4–23.3 m^2^/g with the average values of 18.7 m^2^/g and 13.8 m^2^/g, respectively. Both of the Langmuir and BET specific surface areas have an obvious positive correlation with the TOC content (Fig 4). It indicates that the organic pores play a significant contribution on the specific surface areas in shale samples.

### 4.4 Gas content

Gas logging, gas sorption capacity, desorbed gas content measurement and free gas content calculation were used to qualitatively and quantitatively evaluate the gas content of the Longmaxi Shale. The gas content characteristics of the Longmaxi Shale have been illuminated.

### 4.4.1 Gas logging

Gas logging detected the total hydrocarbon and methane content (vol. %) using a gas logging instrument. From the bottom to top in the Longmaxi Shale Pengye-1 well, both the total hydrocarbon and methane content show decreasing trends. In the bottom part, their values are in the range of 1.45% to 22.75% and 0.94% to 18.45%, with average contents of 8.50% and 6.22%, respectively. In the middle part, their values are in the range of 0.19% to 4.61% and 0.02% to 3.46%, with average contents of 1.53% and 1.1%, respectively. In the top part, their values are in the range of 0.14% to 0.57% and 0.13% to 0.52%, with average contents of 0.28% and 0.21%, respectively. It is obvious that the bottom part is the segment with the highest gas content.

### 4.4.2 Gas sorption capacity

To evaluate the gas sorption capacity of the Longmaxi Shale from Pengye-1 well, an isothermal adsorption experiment was performed using 8 shale samples. The TOC content of these samples ranged from 0.96% to 3.11%. The isothermal adsorption experiment was operated under the Longmaxi Shale temperature (60°C) at moisture equilibration and with a maximum pressure of up to 10 MPa, which slowly increased and had several equilibrium pressure points. The gas sorption capacity of the Longmaxi Shale samples ranged from 0.96 m^3^/t to 3.11 m^3^/t, with an average of 2.05 m^3^/t (Table 4). From the isotherms, the gas sorption content increased rapidly at a relatively low pressure and increased slowly at higher pressures. The rapidly increasing gas sorption was due to the overlapping adsorption between the inner surfaces of pores, with radii slightly larger than that of methane [50].

**Table 4:** Gas sorption capacity, TOC and moisture of the samples from Pengye-1 well.

Depth (m)	TOC (%)	Moisture (%)	Langmuir pressure (MPa)	Langmuir volume (m^3^/t)
2076.9	0.94	1.29	1.88	1.32
2084.85	1.80	2.57	0.92	1.79
2113.46	1.21	3.12	1.25	1.93
2129.21	1.49	1.19	1.47	0.96
2131.80	1.64	1.19	3.69	2.12
2138.13	2.38	2.45	1.68	2.31
2153.42	3.58	2.15	2.24	2.86
2155.14	4.12	2.25	1.45	3.11

**Fig 5:**
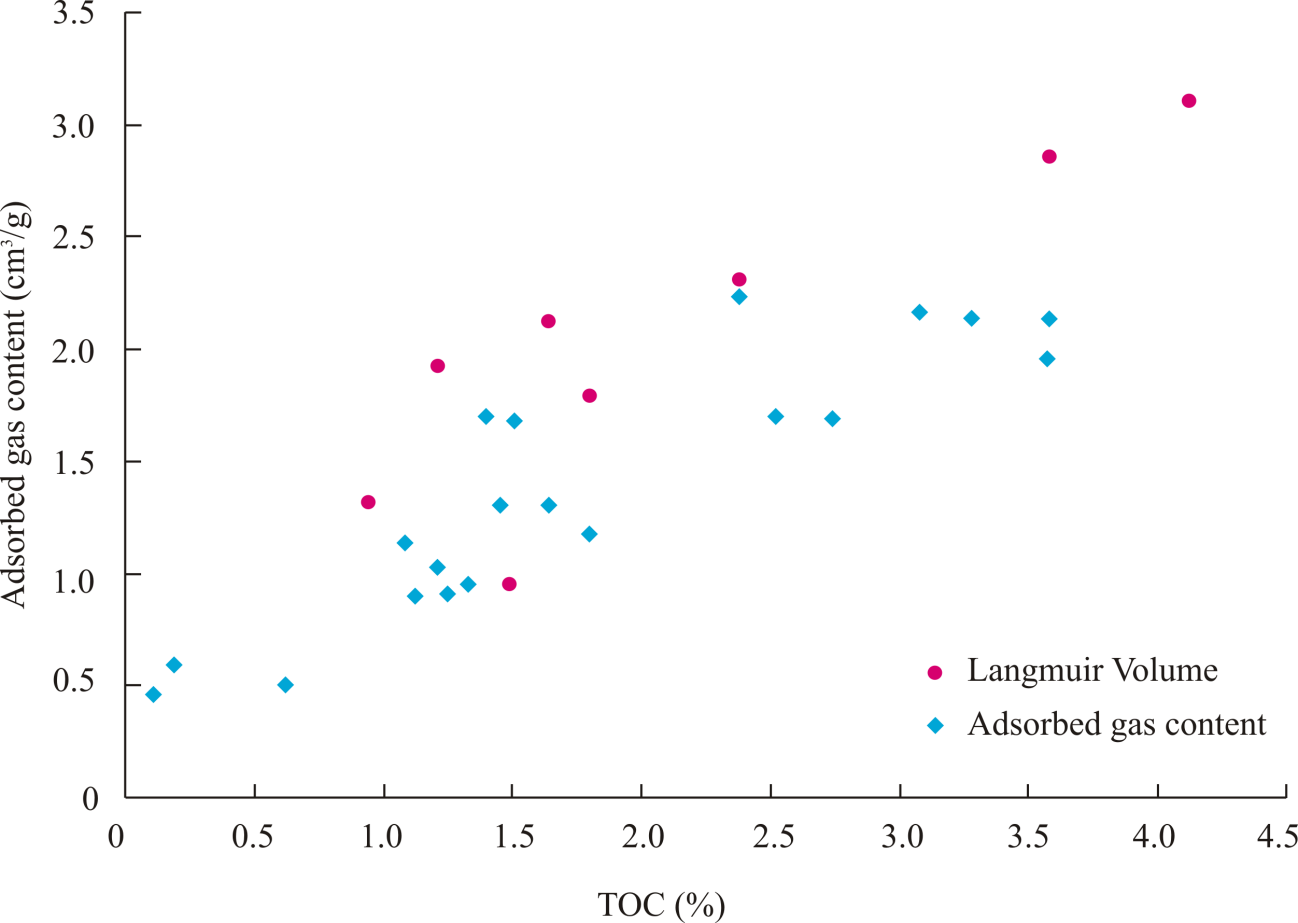
The relationship between the TOC and adsorbed gas content of the samples from Pengye-1 well. (Langmuir Volume is obtained from isothermal adsorption experiment. Adsorbed gas content is got from Desorbed gas content measurement).

The gas sorption capacity increases with increasing of TOC content. This indicates that the bottom of the Longmaxi Shale has the highest gas sorption capacity. There was a positive correlation between the TOC content and Langmuir Volume (*V*_L_) (Fig 5), which represents the gas sorption capacity of the shale sample. This relationship shows that the TOC content is a key factor in controlling the gas sorption capacity of shale samples, which is also consistent with Ross and Bustin (2007). The positive relationship between the TOC content and gas sorption capacity is due to the specific surface area of nano-scale micropores (<200nm), which increases obviously with the increasing of TOC content (Fig 4), created from hydrocarbon generation during formation subsidence and thermal evolution.

### 4.4.3 Adsorbed gas content measurement

The adsorbed gas content of a total of 20 samples ranged from 0.46 to 2.24 cm^3^/g, with an average of 1.38 cm^3^/g including lost, desorbed and residual gas from core samples (110 mm diameter). In the bottom part of the Longmaxi Shale, the gas content of 9 samples ranged from 0.9 to 2.24 cm^3^/g, with an average of 1.8cm^3^/g. In the middle part, the gas content of 9 samples ranged from 0.5 to 1.7 cm^3^/g, with an average of 1.15 cm^3^/g. In the top part, the gas content of 2 samples ranged from 0.46 to 0.59 cm^3^/g, with an average of 0.53 cm^3^/g (Table 5).

**Table 5:** Gas content measurement results of the Longmaxi Shale samples from Pengye-1 well.

Depth	Part of the Longmaxi Shale	TOC (%)	Desorbed gas content measurement					Total porosity (%)	Water saturation (%)	Free gas content (cm^3^/g)
			Desorbed gas content (cm^3^/g)	Lost gas content (cm^3^/g)	Residual gas content (cm^3^/g)	Adsorbed gas content (cm^3^/g)	Core trip time (min)			
2028.60	The top part	0.19	0.0971	0.1159	0.3793	0.5923	121	/	/	/
2031.41		0.11	0.0382	0.0534	0.3689	0.4605	121	1.79	62.52	0.4543
2074.21	The middle part	1.08	0.5915	0.4172	0.1279	1.1366	117	/	/	/
2079.23		1.45	0.6222	0.5722	0.1114	1.3058	117	/	/	/
2081.49		1.51	0.7242	0.7847	0.1751	1.6840	103	3.25	62.74	0.8403
2083.11		1.40	0.7155	0.8499	0.1312	1.6966	94	1.81	55.79	0.5490
2084.27		1.80	0.4580	0.5944	0.1245	1.1769	142	/	/	/
2097.10		0.62	0.2444	0.1301	0.1279	0.5024	113	/	/	/
2113.33		1.21	0.5878	0.3185	0.1173	1.0236	97	3.26	67.17	0.7456
2114.75		1.25	0.4135	0.4156	0.0813	0.9104	97	2.93	66.19	0.6953
2118.65		1.33	0.5572	0.2605	0.134	0.9517	71	/	/	/
2126.73	The bottom part	1.12	0.4402	0.3556	0.1071	0.9029	80	/	/	/
2131.79		1.64	0.7146	0.5494	0.0447	1.3087	81	/	/	/
2135.65		2.74	0.8456	0.7289	0.1165	1.6910	93	/	/	/
2139.85		2.38	1.2422	0.9036	0.0908	2.2366	94	/	/	/
2140.55		3.08	1.1494	0.9017	0.1157	2.1668	94	/	/	/
2147.87		3.28	1.1566	0.8371	0.1377	2.1314	105	/	/	/
2148.37		3.57	0.9102	0.9000	0.1475	1.9577	107	/	/	/
2154.68		3.58	0.9971	0.9590	0.1815	2.1376	112	/	/	/
2157.12		2.52	0.8545	0.7229	0.1248	1.7022	112	4.06	72.33	0.8035

The results also show that the gas content decreases from the bottom to top of the Longmaxi Shale in Pengye-1 well. The Longmaxi Shale has the highest gas content at the bottom part. There is a positive correlation (linear relationship) between the TOC content and gas content (Fig 5), because the specific surface area of nano-scale micropores (<200nm) increases obviously with the increasing of TOC content (Fig 4). This suggests that organic matter plays an important role in shale gas content.

### 4.4.4 Free gas content calculation

Free gas content of Longmaxi Shale in Pengye-1 well can be calculated based on the reservoir pressure, adsorbed gas content, adsorbed phase density, rock bulk density, reservoir temperature, porosity and water saturation by the Formula (4):

$$V_{f}=\frac{P_{r}\cdot T_{s}}{P_{s}\cdot T_{r}}\cdot \frac{1}{\rho_{r}}\left[\Phi \left(1-S_{w}\right)-\frac{V_{a}\cdot \rho_{s}\cdot \rho_{r}}{\rho_{a}}\right]$$ (4)

where *V*_f_ (m^3^/t) is the free gas content per unit mass of shale, *V*_a_ (m^3^/t) is the adsorbed gas content per unit mass of shale, *P*_r_ (MPa) is the reservoir pressure, which is equal to the hydrostatic pressure, *P*_s_ (MPa) is the pressure at standard condition, *T*_r_ (K) is the reservoir temperature, which is range of 59–61°C, *T*_s_ (K) is the temperature at standard condition, *ρ*_r_ (g/cm^3^) is the bulk density of shale sample, *ρ*_s_ (g/cm^3^) is the shale gas density at standard temperature and temperature, *ρ*_a_ (g/cm^3^) is the adsorbed phase density, which is 0.421 (g/cm^3^) as often used for methane adsorbed phase density in coal [50], *Ф* (-) is the pore volume per unit volume shale, *S*_w_ (-) is the water saturation of shale sample.

The free gas content of a total of 6 samples ranged from 0.45 to 0.84 cm^3^/g, with an average of 0.68 cm^3^/g. They are obvious less than adsorbed gas content, and is 24.4–49.7 percent of total gas with an average of 37.5%. These data indicates that adsorbed shale gas is dominant in the Longmaxi Shale of Pengye-1 well.

### 4.4.5 Compare with other shale gas reservoirs

The characteristics of the Longmaxi Shale and other five shale gas reservoirs were listed in Table 6. Compare with the other five shales in America, the Lower Silurian Longmaxi Shale is derived from older sedimentary periods with significantly higher thermal maturity. It indicates that the main gas generation period of the Longmaxi Shale is earlier than those of other five shales and disadvantage for the shale gas enrichment. In addition, after main gas generation period the Longmaxi Shale has experienced several periods of intense tectonic [50], including Indosinian movement and Yanshan movement, which are also unfavorable for the shale gas enrichment.

**Table 6:** Characteristics of the Longmaxi Shale and other five shales (Part data cited from [51, 52]*).

Shale	Basin	Age	TOC (%)	*R*_o_ (%)	Gas content (m^3^/t)	Free gas (%)	Adsorbed gas (%)
Longmaxi	Sichuan Basin	Lower Silurian	0.11–4.34	2.64	0.46–2.24	24.4–49.7	50.3–75.6
Antrim	Michigan Basin	Upper Devonian	0.3–24	0.4–0.6	1.13–2.83	30	70
Ohio	Appalachian Basin	Middle Devonian	0–4.7	0.4–1.3	1.70–2.83	50	50
New Albany	Illinois Basin	Upper Devonian and Lower Mississippian	1–25	0.4–1.0	1.13–2.27	40–60	40–60
Barnett	Fort Worth Basin	Mississippian	4.50	1.0–1.3	8.50–9.91	80	20
Lewis	San Juan Basin	Cretaceous	0.45–2.5	1.6–1.88	0.42–1.27	15–40	60–85

^*^ Data cited by those authors were compiled from Gas Technology Institute/Gas Research Institute research reports and operator surveys.

## 5. Conclusions

The organic and inorganic porosities of these samples are range of 0.08–2.73% and 0.06–2.65%, with the average of 1.10% and 1.76%, respectively. The inorganic pores primarily contribute to the porosity until the TOC content is more than 3%. The inorganic porosities of shale samples with different TOC content are almost equal, while the organic porosities of these samples obviously increase with increasing of TOC content.

Organic matter plays an important role in desorbed gas content. There is a positive correlation between the TOC content and desorbed gas content, because the specific surface area of nano-scale micropores (<200nm) increases obviously with the increasing of TOC content. The adsorbed gas is dominant in the Longmaxi Shale of Pengye-1 well, which ranges from 0.46 to 2.24 cm^3^/g, with an average of 1.38 cm^3^/g. The free gas content ranges from 0.45 to 0.84 cm^3^/g with an average of 0.68 cm^3^/g, and is 24.4–49.7 percent of total gas with an average of 37.5%. The bottom part of the Longmaxi Shale is the most favorable for shale gas exploring, which is higher of brittleness mineral content, porosity and gas content.

Compare with the other five shales in America, the Lower Silurian Longmaxi Shale is derived from older sedimentary periods with significantly higher thermal maturity and has experienced several periods of intense tectonic, which are unfavorable for the shale gas enrichment.
